# Clustering Algorithms: On Learning, Validation, Performance, and Applications to Genomics

**DOI:** 10.2174/138920209789177601

**Published:** 2009-09

**Authors:** Lori Dalton, Virginia Ballarin, Marcel Brun

**Affiliations:** 1 Department of Electrical and Computer Engineering, Texas A&M University, College Station, Texas 77843-3128, USA; 2 Facultad de Ingenieria, Universidad Nacional de Mar del Plata, Mar del Plata, Argentina

**Keywords:** Clustering, genomics, profiling, microarray, validation index.

## Abstract

The development of microarray technology has enabled scientists to measure the *expression *of thousands of genes simultaneously, resulting in a surge of interest in several disciplines throughout biology and medicine. While data clustering has been used for decades in image processing and pattern recognition, in recent years it has joined this wave of activity as a popular technique to analyze microarrays. To illustrate its application to genomics, clustering applied to genes from a set of microarray data groups together those genes whose expression levels exhibit similar behavior throughout the samples, and when applied to samples it offers the potential to discriminate pathologies based on their differential patterns of gene expression. Although clustering has now been used for many years in the context of gene expression microarrays, it has remained highly problematic. The choice of a clustering algorithm and validation index is not a trivial one, more so when applying them to high throughput biological or medical data. Factors to consider when choosing an algorithm include the nature of the application, the characteristics of the objects to be analyzed, the expected number and shape of the clusters, and the complexity of the problem versus computational power available. In some cases a very simple algorithm may be appropriate to tackle a problem, but many situations may require a more complex and powerful algorithm better suited for the job at hand. In this paper, we will cover the theoretical aspects of clustering, including error and learning, followed by an overview of popular clustering algorithms and classical validation indices. We also discuss the relative performance of these algorithms and indices and conclude with examples of the application of clustering to computational biology.

## 1. INTRODUCTION

Microarray technology has made available an incredible amount of gene expression data, driving research in several areas including the molecular basis of disease, drug discovery, neurobiology, and others. Usually, microarray data is collected with the goal of either discovering genes associated with some event, predicting outcomes based on gene expression, or discovering sub-classes of diseases. While clustering has been used for decades in image processing and pattern recognition [1-3], in recent years it has become a popular technique in genomic studies for extracting this kind of valuable information from massive sets of gene expression data.

Clustering applied to genes from microarray data groups together those whose expression levels exhibit similar behavior through the samples. In this context, similarity is taken to indicate possible co-regulation between the genes, but may also reveal other processes that relate their expression. In other words, the application of clustering in our first goal listed above is founded by the concept of “guilty by association”, where genes with similar expression across samples are assumed to share some underlying mechanism.

On the other hand, clustering applied to samples may help with our second and third goals, since when used this way it offers the potential to discriminate pathologies, or other conditions, based on their differential patterns of gene expression. It may also provide clues to the existence of previously undetected groupings within the samples, or be used to detect sub-groups in a non supervised manner.

As a short historical review, we cite a few key reference papers. In 1997, Somogyi *et al*. [4] applied the Fitch-Margoliash clustering algorithm, used previously on phylogenetic trees, to display genes grouped by similarity in their wiring and behavior (network trajectories). Subsequently, Eisen *et al*. [5] described the use of *hierarchical* clustering, combined with a color representation of the expression intensity, to group and visualize genes with similar *profile*, or expression patterns. In 1999, Ben-Dor, Shamir and Yakhini proposed a new algorithm based on graph theory called CAST and the visualization of the distance matrix as an intensity matrix [6]. In the same year, we can find the works of Goulub [7] and Tamayo [8] *et al*. which suggested the use of self organizing maps (SOM) as a clustering algorithm for gene expression, and the work of Tavazoie *et al*. [9], which used the K-means algorithm to identify transcriptional regulatory sub-networks. Another graph based algorithm called CLICK was introduced in 2000 by Sharan and Shamir [10]. In 2001, Yeung *et al*. [11] presented the use of model based clustering, where the clusters are modeled as mixtures of Gaussian distributions, and proposed the use of the BIC criterion for selecting the number of clusters. Dougherty *et al*. presented in 2002 [12] an algorithm to select the best clustering rule for a dataset, based on noise injection, replication, and cluster accuracy. In 2003, Dembele and Kastner [13] described a modified fuzzy c-means algorithm applied to genomic data, which automatically selects the fuzziness parameter. Finally, the use of nonnegative matrix factorization (NMF) was introduced in 2004 by Brunet *et al*. [14], with the intent to alleviate some of the disadvantages of other clustering techniques. A few review papers on clustering algorithms applied to microarray data can also be found in the literature, describing the advantages and shortcomings of each algorithm and sometimes including validation techniques in their analysis [15, 16].

Although used for many years in the context of gene expression microarray data, clustering has remained highly problematic [2, 12, 17]. Some criticisms raise the question as to whether clustering can be used for scientific knowledge [18]: how may one judge the relative worth of clustering algorithms unless the assessment is based on their inference capabilities? Although the ability of clustering algorithms to make inferences has been addressed to some extent, a mathematical foundation for clustering has been provided only very recently [19, 20].

In this paper we will cover a mathematical model of clustering and review learning in section 2. We provide an overview of popular clustering algorithms in section 3 and validation indices in section 4, followed by a discussion on the relative performance of these algorithms and indices in section 5. Finally, we comment on the application of clustering to genomics in section 6.

## 2. MATHEMATICAL MODEL OF CLUSTERING

In the context of pattern recognition theory, each object is represented by a vector of features, called a pattern. *Clustering* can be defined as the process of partitioning a collection of vectors into subgroups whose members are similar relative to some distance measure. A *clustering algorithm* receives a set of vectors, and groups them based on a cost criterion or some other optimization rule.

The related field of pattern classification, which involves simply assigning individual vectors to classes, has developed a theory based on defining error criteria, designing optimal classifiers, and learning. In comparison, clustering has historically been approached heuristically; there has been almost no consideration of learning or optimization, and error estimation has been handled indirectly *via *validation indices. Only recently has a rigorous clustering theory been developed in the context of random sets [19]. Although we will not go over the mathematical details of [19, 20], in this section we summarize some essential points regarding clustering error, error estimation, and inference.

### 2.1. Model

Within a probabilistic framework, objects to be clustered are assumed to be described by vectors of numerical values. These vectors are realizations of a random labeled point process, which produces random sets in a multi-dimensional space with unknown random labels associated with each vector. Two vectors are properly in the same cluster if and only if they have the same label produced by the random process [19]. Thus, a clustering algorithm may be viewed as an operator on random sets which partitions their elements into groups by assigning labels to them.

The *labeling error* of a clustering algorithm is the expected number of discrepancies between the labels it assigns and the true labels generated by the labeling process. Since the disagreement between two partitions should not depend on the indices used for the labels, or the names assigned to each cluster, we define the *partitioning error* to be the minimum of the labeling errors for all possible permutations of the labels. The partitioning error applies to a specific realization of a random point labeling process, so we define the *clustering error* (also called the *misclassification error*) of a clustering algorithm with respect to a process to be the expected value of the partitioning error.

Estimation of the clustering error is done in the usual manner to assess the performance of a cluster operator applied to a given random process model. We generate independent synthetic data consisting of a collection of sets, or samples, representing realizations of the process, apply the clustering algorithm to the samples, compute the partitioning error for each realization of the cluster labels against the true partitions, and average over the realizations to obtain an estimate of the clustering error [12]. See [19] for a more complete and formal presentation on the probabilistic model.

### 2.2. Learning

Clustering is usually considered to be the problem of partitioning a single set of unlabeled points. All of the traditional algorithms covered in section 3 (K-means, fuzzy c-means, hierarchical clustering, etc.) fall in this category. In this setting, several clustering algorithms may be considered, and the election of one could be based on their performance on some internal or relative index. However, we may view the problem of clustering or the selection of a best clustering algorithm more generally as a problem of learning. Consider observing training data consisting of a collection of *N* training sets, including their labels, where each set is a realization of a point process and their label functions are realizations of a random labeling process. A *clustering rule* maps this given training data of size *N* to a label operator, which induces a cluster operator that should approximate the partitions created by the labeled point process.

As with classification rules, both the label operator and the cluster operator are random because they depend on the training data. Also, as with classification where the investigator may choose between different rules, the burden is on the investigator to choose or develop a suitable clustering rule to design the cluster operator from the training data. We may proceed with an approach analogous to learning in classification theory [21], by first treating learning in the binary setting and subsequently extending to an arbitrary finite label set. There has been some previous work in learning clustering algorithms in the context of learning by examples. For instance, researchers have applied learning for determining a metric of the space, learning similarity matrices for spectral clustering, utilizing local structure, and applying nonparametric methods [22-25].

In classification, the simplest way to apply learning is to select from a set of classifiers the one with the lowest expected error for a particular model. Similarly, the selection of the best clustering algorithm can be done by empirical risk minimization [21]: given training data consisting of realizations from a labeled point process, and given a collection of clustering operators or algorithms, the best operator should minimize the clustering error on the training data. Therefore, if the underlying random labeled point process is unknown but labeled samples are available, it is possible to select a cluster operator that will be optimal relative to the family of operators analyzed.

Minimizing empirical risk is just a simple way to train from sample data. In the case of classification, a more efficient method is to apply *rules* to design classifiers from the sample data, optimizing some predefined criteria. Examples are the *k*NN, LDA, perceptron, and SVM classification rules. The same approach is possible in clustering—we could create a clustering rule designed to generate clustering operators that optimize some criteria based on the training data, and the behavior and design of these operators would be totally different from any of the currently popular clustering algorithms. However, while learning in clustering may be viewed analogously to classification in this way, it presents a much more complex problem since individual training points sampled from a distribution in a *D*-dimensional space are each replaced by entire sets drawn from a labeled point process.

An example of this approach adapts the *k*NN classification rule to an analogous clustering rule. When used in classification, the popular and non-parametric *k*-nearest neighbor algorithm [26] decides the label of a new sample based on a majority vote from the labels of the *k* closest, or most similar, training points. Work in [19, 27] proposes a similar learning algorithm for clustering, where the training samples are now labeled sets. Given a new set we wish to cluster, we find the *k* most similar objects in the training data based on Hausdorff distance, and use the labels of the chosen sets to define a labeling, and therefore a clustering, for the new set.

An example of the performance of a trained clustering algorithm is provided in section 5.2, and additional details and theory can be found in [19, 27].

## 3. CLUSTERING ALGORITHMS

Clustering algorithms may be categorized by how they form groups of clusters. *Hierarchical algorithms* work on either successive splitting (*divisive*) or merging (*agglomerative*) of groups to form a hierarchy of clusters based on a specified measure of distance or similarity between objects. Alternatively, *partitioning algorithms* search for a partition of the data that optimizes a global measure of quality for the groups, usually based on distance between objects.

Hierarchical algorithms may also be subclassified by the way the distances or similarities between objects are updated after splitting or merging groups (*linkage*), which has a significant influence on the structure of the resulting clusters. Hierarchical algorithms are also extensively used to generate multiple partitions of the data, since each level of the hierarchy is a different partition of the data.

Another way to classify clustering algorithms is based on their output: in *hard clustering* the output is a partition of the data, while in *soft clustering* (i.e., fuzzy clustering) the output is a membership function, so each pattern can belong to more than one group with varying degrees of membership. A fuzzy cluster defines a natural hard partition of the data from the maximum membership of each object. Fig. (**1**) shows a simple taxonomy of clustering algorithms [16].

The selection of a particular algorithm should be based on considerations of the problem at hand, including the acceptable level of error, the amount of computational resources available, the type of clusters required, and needs on visual representation. Each algorithm has its own strengths and weakness and may be better suited than other algorithms for particular tasks. For example, hierarchical clustering algorithms are especially useful for exploratory data analysis because they do not need prior specification of the number of clusters and their outputs can be visualized in a tree structure.

Most partitioning algorithms are based on the minimization of an objective function, which is a measure of the quality of a partition of the data. The most common objective function is the average of the squared distance to the centroid of the clusters [2], as in the K-means algorithm described in section 3.1. This particular objective function may be interpreted as a measure of how good the centers are as representatives of the clusters. Though it works well with similarly sized compact clusters, it is limited by the need of each cluster center to represent its points and often fails when the shape of a grouping is more complex or when there is a large difference between the number of points in each cluster. A second major drawback of this objective function is that it decreases with the number of clusters in a nested sequence of partitions, favoring a large number of small clusters.

Most clustering algorithms are designed with an iterative approximation approach which starts with an initial seed partition and successively modifies it to reduce the objective function in each step. This process is repeated until no change is observed or the algorithm reaches a stop criterion.

The following reference list is not exhaustive, though other algorithms used for clustering gene expression data are usually variations of these. Many good references regarding clustering algorithms and their mathematical and algorithmic aspects are available [28, 2, 29].

### 3.1. K-Means

One of the most common iterative algorithms is the K-means algorithm [2, 29], broadly used for its simplicity of implementation and convergence speed. K-means also produces relatively high quality clusters considering the low level of computation required.

The algorithm is presented with a set of *n* sample vectors and a number *K* for the expected number of clusters, and produces *K* centroids that attempt to minimize the objective function, which is the average distance of each sample vector to their nearest centroid. A typical implementation of the algorithm starts with a random election for the centroids, iteratively assigns each vector to the nearest centroid, and updates the new centroid positions until convergence is reached [30, 31].

K-means is one of the simplest algorithms known to perform well with many data sets, but its good performance is limited mainly to compact groups. When the points are drawn from a mixture of Gaussian distributions, the K-means algorithm is a gradient descent algorithm that minimizes the quantization error [32]. As with many gradient descent algorithms, one drawback of K-means is that it can reach a local minimum of the objective function instead of the desired global minimum, meaning that convergence is reached but the solution is not optimal. An analysis of this problem is presented in Dougherty *et al*. [12]. One way to overcome this is by running the algorithm multiple times with different random seeds and selecting the partition that appears with the highest frequency.

### 3.2. Fuzzy C-Means

In the K-means algorithm, each vector is classified as belonging to a single cluster (hard clustering), and the centroids are updated based on the classified samples. In a variation of this approach known as fuzzy c-means [2, 29], all vectors have a degree of membership for each cluster, and the respective centroids are calculated based on these membership degrees.

Whereas the K-means algorithm computes the average of the vectors in a cluster as the center, fuzzy c-means finds the center as a weighted average of all points, using the membership probabilities for each point as weights. Vectors with a high probability of belonging to the class have larger weights, and more influence on the centroid.

As with K-means clustering, the process of assigning vectors to centroids and updating the centroids is repeated until convergence is reached.

### 3.3. Hierarchical

Hierarchical clustering [2] creates a hierarchical tree of similarities between the vectors, called a dendrogram. The usual implementation is based on agglomerative clustering, which initializes the algorithm by assigning each vector to its own separate cluster and defining the distances between each cluster based on either a distance metric (e.g., Euclidean) or similarity (e.g., correlation). Next, the algorithm merges the two nearest clusters and updates all the distances to the newly formed cluster *via *some linkage method, and this is repeated until there is only one cluster left that contains all the vectors. Three of the most common ways to update the distances are with *single*, *complete* or *average* linkages.

This process does not define a partition of the system, but a sequence of nested partitions, where each partition contains one less cluster than the previous partition. To obtain a partition with *K* clusters, the process must be stopped *K* − 1 steps before the end.

Different linkages lead to different partitions, so the type of linkage used must be selected according to the type of data to be clustered. For instance, complete and average linkages tend to build compact clusters, while single linkage is capable of building clusters with more complex shapes but is more likely to be affected by spurious data [12].

### 3.4. Expectation Maximization

Expectation maximization clustering [11, 33] estimates the probability densities of the classes using the Expectation Maximization (EM) algorithm. The result is an estimated set of *K* multivariate distributions each defining a cluster, with each sample vector assigned to the cluster with maximum conditional probability.

Different assumptions on the model correspond to different constraints on the covariance matrices of each distribution. Examples of these constraints are modeling spherical *vs*. elliptical densities and using the same *vs*. different prior probabilities. The less strict the constraints the more flexible the model, but at the same time more samples would be necessary for good estimates of the additional parameters. As always, the user of the algorithm is responsible for deciding the level of constraints to apply based on the amount of data available.

### 3.5. Self Organizing Maps

By applying self organizing maps (SOM) to the data, clusters can be defined by points on a grid adjusted to the data [34, 35]. Usually the algorithm uses a 2-dimensional grid in a higher dimensional space, but for clustering it is typical to use a 1-dimensional grid.

SOM clustering is very useful in data visualization since the spacial representation of the grid, facilitated by its low dimensionality, reveals a great amount of information on the data.

## 4. VALIDATION INDICES

Historically, a host of “validity” measures have been proposed for evaluating clustering results based on a single realization of a random-point-set process [36-41]. No doubt one would like to measure the accuracy of a cluster operator based on a single application. But is this feasible? Clearly it would be absurd to assess the validity of a classifier based on a single point without knowledge of the true label of that point. This is analogous to evaluating the validity of a cluster operator on a single point set without knowledge of the true partition, but there is a difference that provides hope. The output of a cluster operator consists of a partition of a point set with a spatial structure, and one can define measures for different aspects of this structure, for instance, compactness. It could be hoped that such measures assess the *scientific validity* of a clustering algorithm, and for a validity measure to assess scientific validity, *ipso facto*, it must be closely related to the error rate of the cluster operator as that rate is defined within a probabilistic theory of clustering.

Validity measures proposed for clustering algorithms fall broadly into three classes. The first type is based on calculating properties of the resulting clusters, such as compactness, separation and roundness. This approach is called *internal validation* because it does not require additional information about the data. A second approach is based on comparisons of partitions generated by the same algorithm with different parameters or subsets of the data. This is called *relative validation* and also does not require additional information. The third approach, called *external validation*, compares the partition generated by the clustering algorithm to the true partition of the data. External validation corresponds to a kind of error measurement, either directly or indirectly. Therefore, we should expect external methods to be better correlated to the true error [20], although they cannot be measured in practice.

In this section, we will cover several internal, relative, and external validation indices, discuss their motivation, and overview some of their advantages and disadvantages.

### 4.1. Internal Validation Indices

Internal validation is the simplest way to evaluate a clustering algorithm applied to a data set, since it uses only the spacial distribution of the points and the cluster labels generated by the algorithm to compute properties of the clusters. This family of techniques is based on the assumption that the algorithms should search for clusters whose members are close to each other and far from members of other clusters. Some of these indices are described below.

#### 4.1.1. Dunn’s Indices

*Dunn’s validation index* is conceptually the simplest of the internal validation indices: it compares the size of the groups with the distances between groups. The further apart the groups, relative to their size, the larger the index and the “better” the clustering. This index, *V*(***C***), is computed as the ratio between the minimum distance between two clusters and the size of the largest cluster [42-44]:

(1) $$V\left(C\right)=\frac{\min_{h\neq k}d_{c}\left(C_{k},C_{h}\right)}{\max_{k}\Delta \left(C_{k}\right)}$$

where *d_C_*(*C_k_*,*C_h_*) is the distance between two clusters (or *linkage*) and Δ(*C_k_*) is the size of the cluster *C_k_*. There are many methods for computing both *d_C_*(*C_k_*,*C_h_*) and Δ(*C_k_*), and every combination of these defines a different Dunn’s index. For example, five measures for distance between clusters (linkage) are the single, complete, average, average to centroids, and Hausdorff metrics [44], and three possible measures for cluster size are the complete, average, and centroid diameters [44]. More formal definitions of these cluster distances and sizes can be found in [20].

The ability to mix and match distance and cluster-size measures grants a large number of options that can be overwhelming to the user. Usually, the combination of average to centroid distance and centroid cluster size provides an index simple to understand: compact circular clusters with well separated centers tend to produce a higher Dunn’s index. Another useful pair are the complete distance and complete size measures [20].

#### 4.1.2. Silhouette Index

The *silhouette* of a cluster was first introduced as a display technique to evaluate visually which points lie well inside the cluster and which do not [45], based on the *silhouette width* of their points.

The silhouette width of a given point defines its proximity to its own cluster relative to its proximity to other clusters. Mathematically, the silhouette width for each point **x** is defined by

(2) $$S\left(\text{x}\right)=\frac{b\left(\text{x}\right)-a\left(\text{x}\right)}{\max\left[b\left(\text{x}\right),a\left(\text{x}\right)\right]}$$

where *a*(**x**) is the average distance between **x** and all other points in its cluster, and *b*(**x**) is the minimum of the average distances between **x** and the points in the other clusters.

The silhouette of a cluster is defined as the average silhouette width of its points. Finally, aggregating information from all points, the *global silhouette* index for a whole clustering partition is the average silhouette of the clusters [43, 44].

For a given point **x**, its silhouette width ranges from −1 to 1. If the value is close to −1, the point is on average closer to another cluster than the one to which it belongs. If the value is close to 1, then its average distance to its own cluster is significantly smaller than that to any other cluster. Thus the higher the silhouette, the more compact and separated the clusters.

In an interesting application to genomics, the silhouette index was used successfully for SNP genotype calling [46] to evaluate the separation of the clouds of points obtained by the measurement of many samples.

### 4.2. Relative Validation Indices

While internal indices evaluate a single realization of the clustering algorithm, they can not sense the stability of the algorithm against variations in the data, or consistency of the results in the case of redundancy. A family of more complex indices, called *relative validation* indices, attempts to measure the consistency of an algorithm by comparing the clusters obtained by the same algorithm under different conditions. These indices often attempt to exploit redundancy in the data.

#### 4.2.1. Figure of Merit

The *figure of merit* (*FOM*) index [47] assumes redundancy is imbedded in the sample data to measure consistency. As an example, consider a clustering algorithm applied to microarray data, which should generate clusters representing different biological groups. Therefore, samples in the same cluster should possess similar pattern vectors (expression profiles) for additional features that were not considered in the clustering process. If we ignore a feature (gene) when we cluster, will the resulting partition still have nice properties in the neglected dimension?

The FOM of a feature is computed by clustering the samples after removing the given feature and measuring the size of these clusters (i.e., the average distance between all samples and their cluster’s centroids) specifically on the feature under inspection. If this average distance is small, then the clustering algorithm partitioned the samples in compact clusters even with the feature removed, suggesting that the algorithm is consistent. The overall FOM for a clustering algorithm is the sum of these values over all features, leaving each one out one at a time.

Heuristically speaking, a clustering algorithm that produces consistent clusters should be able to predict removed features, and therefore would have a low FOM index. However the FOM performance based on simulated data is usually below that of simpler validation indices, including many internal ones. This fact, coupled with the necessity of repeating clustering many times, makes this measure one of the last choices for selecting a validation index [20].

#### 4.2.2. Stability

The instability index measures the ability of a clustered data set to predict the clustering of another data set sampled from the same source [48].

Stability is measured by first dividing the set of points to be clustered in two parts. The clustering algorithm under examination is applied to the first part, and the labels obtained over these points are used to train a classifier that partitions the whole space. Both the original clustering algorithm and this classifier are applied to the second collection of points, generating two sets of labels. The disagreement between these labels, averaged over repeated random partitions of the points, defines the *instability* of the clustering algorithm.

The instability index depends on the number of clusters, and therefore needs to be normalized when used for model selection [48]. The normalization is obtained by dividing by the instability obtained when using a random estimator as the classifier.

Another issue affecting the instability index is the selection of the classification rule, which can strongly influence the results [48, 20]. Also, simulation studies show that this index does not perform better than other relative and internal indices, though it is one of the most time consuming indices since it involves the repeated application of a clustering algorithm and training a classifier.

### 4.3. External Validation Indices

The last family of indices, called *external validation* indices, compares properties of an algorithm’s proposed clusters against that of known true clusters.

#### 4.3.1. Hubert’s Correlation

The Hubert Γ statistic measures the correlation between the co-occurrence matrices (a matrix with entry *I*(*i*, *j*) = 1 if objects *i* and *j* belong to the same cluster and *I*(*i*, *j*) = 0 otherwise) of both the expected partition and the one obtained by applying a clustering algorithm [39]. It essentially exploits the notion that similar partitions have similar co-occurrence matrices, which in turn will have a high correlation.

One great advantage of this measure is that it does not rely on permutations of the labels, since the co-occurrence matrices are independent of the labels used to define the partitions. Furthermore, simulation studies have reported good performance for this measure when predicting the error of the clustering algorithm [20].

#### 4.3.2. Rand Statistics, Jaccard Coefficient, and the Folkes and Mallows Index

These measures analyze the relationship between pairs of points using the co-occurrence matrices for the expected partition and the one generated by the clustering algorithm [39]. For a given pair of points, **x** and **y**, there are four possibilities: (a) **x** and **y** fall in the same cluster in both the expected and the computed partition, (b) **x** and **y** fall in the same cluster in the computed partition, and in different clusters in the expected partition, (c) **x** and **y** fall in the different clusters in the computed partition, but in the same cluster in the expected partition, or (d) **x** and **y** fall in different clusters in both the expected and the computed partition.

The measure of disagreement between the partitions is quantified by the number of pairs of points that fall in each category. Let *a*, *b*, *c*, and *d* be the numbers of pairs of different points that belong to situations (*a*), (*b*), (*c*), and (*d*), respectively, and let *M* = *n*(*n* − 1)/2 be the number of pairs of different points. Some indices to measure the agreement between the partitions based on these values are the *Rand statistic*, defined by *R* = (*a* + *d*)/*M*, the *Jaccard coefficient*, defined by *J* = *a*/(*a* + *b* + *c*), and the *Folkes and Mallows index*, defined by $\mathrm{FM}=\sqrt{\left(a/\left(a+b\right)\right)\left(a/\left(a+c\right)\right)}$.

The Rand statistic measures the proportion of pairs of vectors that agree by belonging either to the same cluster (*a*) or to different clusters (*d*) in both partitions. The Jaccard coefficient measures the proportion of pairs that belong to the same cluster (*a*) in both partitions, relative to all pairs that belong to the same cluster in at least one of the two partitions (*a* + *b* + *c*). Finally, the Folkes and Mallows index measures the geometric mean of the proportion of pairs that belong to the same cluster in both partitions (*a*) relative to the number of pairs that belong to the same cluster for at least one partition, (*a*+*b* and *a*+*c*). In simulation studies these measures perform well at predicting misclassification rate [20].

## 5. COMPARATIVE ANALYSIS

In sections 5.1 through 5.3, we review results from several comparative studies on the performance of clustering algorithms and validation indices. We also include results from a new simulation study comparing the general performance of classical clustering algorithms in section 5.4.

### 5.1. Effect of Replicates on Clustering Error

The goal of earlier work in [12] was to study how replication, or repeated realizations of an experiment, could be used to reduce the error of clustering algorithms when applied to microarray data. The results were displayed as error graphs, giving the misclassification rate as a function of the number of replicates. Replicated data was averaged to reduce variability, simulating technical or biological replicates used in microarray based experiments.

The amount of replication was varied from 1 to 20 replicates. The algorithms tested were K-means, fuzzy c-means, SOM and hierarchical (with both Euclidean distance and correlation distance metrics). The basic design of each experiment was to generate synthetic data with distributions obtained from real microarray data, vary the amount of dispersion (variance) of the data, and measure the error of the algorithms under the different amounts of replication.

The article in [12] showed how the misclassification rate, later formalized in [19], could be used to assert the performance of several algorithms under conditions similar to the ones prevailing in microarray studies. Unsurprisingly, the results demonstrated that lower variance and more replications tend to yield greater precision. It also provided a model to evaluate and select the best algorithm for a particular data set and find the amount of replication needed.

Fig. (**2**) shows examples of the results of this analysis. Example (a) illustrates how a low number of replicates results in a large number of clustering misclassifications, while in example (b) a larger number of replicates improves performance for the same clustering algorithm.

### 5.2. Using Learning to Improve Performance

In [19, 27] the authors have presented a probabilistic theory of clustering, including error estimation (testing) and learning (training). As discussed in section 2.2, their key insight is that while classification theory designs operators on random variables, the theory of clustering should be based analogously on operators over random sets. Thus the clustering problem is best viewed as a problem of learning: given a collection of labeled training data, where each training sample is now a whole set of data drawn from a labeled point process rather than a single labeled point from a random variable, we aim to design a cluster operator that can use this training data effectively to estimate the partitions for any new data sets we observe that are drawn from the same point process.

In these papers, the authors compare the *k*NN clustering rule described in section 2.2 to traditional clustering algorithms. They considered several models for the clusters and showed that for a sufficiently large number of training sets, the performance of trained cluster operators surpasses that of all classical clustering algorithms considered. An example is provided in Fig. (**3**), where error rate is graphed as function of the number of training samples. In this example, the random sets were generated by Gaussian and circular distributions with random translations. Without learning, the Fuzzy C-means algorithm clearly outperforms the other classical algorithms, but they all are outperformed by the trained cluster operator when enough training sets are provided.

### 5.3. Association of Indices with Error Measure

In [20], a number of proposed validity measures were examined as to how well they correlate with clustering error across a number of clustering algorithms and random-point-set models. To quantify the degree of similarity between the validation indices and the clustering errors, we used Kendall’s rank correlation between their values.

The results indicated that, overall, the performance of validity indices is highly variable. For complex models or when a clustering algorithm yields complicated clusters, both the internal and relative indices fail to predict the error of the algorithm. Some external indices appeared to perform well, whereas others did not.

It was concluded that one should not put much faith in a validity score unless there is evidence, either in terms of sufficient data for model estimation or prior model knowledge, that a validity measure is well-correlated with the error rate of the clustering algorithm.

Fig. (**4**) shows examples of scatter plots between a few validation indices and the misclassification error [20]. In this figure, we can see how bad the correlation is between the clustering error and both the FOM (c) and stability (d) indices. It also shows differences between external indices: for this model the Rand statistic (e) is excellent with a one to one relationship with error, but the Folkes and Mallows index (f) starts to disagree with the error when the error is large.

### 5.4. Performance of Classical Clustering Algorithms

In this section we compare several classical clustering algorithms relative to three measures of performance: memory usage, computational time, and error rate. The algorithms considered in this study are K-means (KM), fuzzy c-means (FCM), hierarchical with the Euclidian distance metric and complete, single, and average linkages (HCEc, HCEs, and HCEa, respectively), hierarchical with the centered correlation based metric and complete linkages (HCC), SOM with a 1-dimensional grid using Euclidean distance and Gaussian type neighbors, and the EM algorithm with an equal spherical variance model.

Our random process model uses simple Gaussian distributions for each cluster, with the number of samples per cluster as constant as possible. We use a fixed mean vector template model for the centers of the clusters, meaning that the center of each cluster is always fixed in the same location for all realizations of the random point process. Furthermore, each center was placed on a 2 dimensional grid. For example, 8 classes were represented with the centers of each cluster on a 2×4 rectangle, 16 classes by a 4×4 square, and 32 classes by a cross-shaped pattern extending the 4×4 square. In this way, as we increase the number of clusters we are simply expanding the number of nodes used in this grid. The same grid is used in all experiments regardless of the number of dimensions, thereby giving comparable results between simulations with different numbers of clusters and dimensions.

We report how our three measures of performance scale with the complexity of the problem to solve (the number of dimensions, samples, clusters, and the variance of each cluster). Each experiment was defined by a cluster model with parameters selected from the following options:

- Number of dimensions: *D* = 2, 4, 8, 16, 32, 64, 128
- Total number of points: *n* = 50, 100, 200, 500, 1000, 2000, 5000
- Number of clusters: *K* = 2, 4, 8, 16, 32
- Variance within each cluster (compactness): σ^2 ^= 0.1, 0.25, 0.5, 1, 2.5, 5

These results provide some insight on the strengths and weaknesses of each algorithm, and their suitability in different experimental conditions.

#### 5.4.1. Maximum Memory Usage and Computational Time

To study memory usage, we examined our source code for each algorithm and calculated the maximum memory allocated to defined variables. We exclude from the analysis any input and output memory requirements that all algorithms have in common, namely the input data (an *n*×*D* array), the number of samples (*n*), the number of dimensions (*D*), the desired number of clusters (*K*), and the output cluster labels (a length *n* vector). We assume 4 bytes are required for integers and 8 bytes for floating point variables. To report average computational time, all experiments were executed on similar computers to obtain comparable results.

Our implementation of FCM uses KM to initialize the clusters, and EM calls FCM to initialize clusters, which adds a bit of time and memory to each of these algorithms. A summary of our memory results is presented in Table **1**, along with examples from a representative selection of experiments. Run times for the same experiments and the corresponding values of *K*, *D* and *n* used in each experiment are shown in Table **2** for comparison.

The SOM algorithm usually requires the smallest amount of memory, which is independent of the number of samples, *n*. The KM and SOM algorithms are consistently among the fastest algorithms, and they are especially fast when there are a large number of clusters. See for example experiment 2 in Table **2**. However, we will see in the next section that the clustering error of these algorithms can be relatively high in this case. In high dimensions, the KM, SOM, and FCM algorithms have comparable running times, although FCM is more sensitive to the number of clusters.

The EM algorithm tends to have high memory requirements to hold *nK* conditional probabilities and is generally a middle performer with erratic results in terms of computational time. This unpredictability may be exacerbated in our implementation, which occasionally starts over to avoid assigning empty clusters.

Memory allocation for the HC algorithm only depends on *n*, and most notably it defines an *n*×*n* sample distance matrix and a size 3×*n* linkage tree matrix. Although usually the slowest algorithm, not surprisingly it has a very predictable execution time which depends almost entirely on the total number of samples for all clusters. This is because it always builds the same sized tree no matter the nature of the samples it is trying to cluster.

#### 5.4.2. Clustering Error Results

In this section, we report the clustering error between the generated cluster labels and the true classes, as defined in section 2.1. These results are provided in four sets of graphs. Fig. (**5**) contains 12 plots of the error rate (*y*-axis) against the variance of each cluster (*x*-axis), with each plot representing a different fixed set of values for the parameters *K*, *D*, and *N*. These best illustrate the effect of increasing the variance of the clusters in various scenarios. Similarly, Figs. (**6-8**) each contain several plots of the misclassification error against the number of classes, dimensions, and samples, respectively. All plots use a log scale on the *x*-axis.

At the highest levels of variance where the true clusters are highly mixed, for example with σ^2 ^= 2.5 and σ^2 ^= 5, there is not much any algorithm can do to discern the classes. Performance for all algorithms tends to converge to that of a random cluster label assignment, which may be considered a baseline for comparison and is represented by thick solid lines in our plots.

For *K* = 2, *D* = 2, and *n* = 100, see the upper left plot in Fig. (**5**). At low variance, the best algorithms are FCM, SOM, KM, and EM, followed by HCEc and HCEa, and finally the HCEs and HCC algorithms trail behind. At higher variances, the EM algorithm does a little more poorly than FCM, SOM, and KM. Meanwhile, HCC starts to improve and beats the Euclidean based HC algorithms to approach the performance of the FCM, SOM, and KM algorithms. Generally we’ve found that using the current model and excepting very extreme cases, the FCM algorithm tends to have the lowest misclassification error with the KM, SOM, and EM algorithms also performing competitively.

#### 5.4.3. Clustering Error Discussion

Our study revealed advantages and disadvantages for each clustering algorithm we considered. The following discussion compares algorithms and covers our observations in detail to give a general idea on which algorithm might be the most suitable for a given set of experimental parameters.

At least for simple Gaussian distributed models, it’s hard to find a scenario where the HCEs algorithm should be used. In the majority of cases we simulated, it has a higher error rate than random cluster labels and should only be considered in very low variances. For example, in the left column of Fig. (**6**) it achieves zero error for all *K*, along with all the other Euclidean distance based hierarchical algorithms, while KM, SOM, FCM, and EM have increasing errors as the number of clusters grows. However in the second and third columns of the same figure, which consider the same parameters with increased variance, the HCEs algorithm often performs even worse than random labels, especially when there are a small number of clusters. It should be noted that with more complex cluster shapes and arrangements, the KM, FCM, HCEc, and other usual methods may fail to discriminate the clusters, while the HCEs algorithm with single linkage may have an advantage (in low noise) because it is the only one that does not look for circularly shaped clusters.

With extremely low variances (σ^2 ^= 0.1 or less—in this case the clusters are almost not overlapping), the HCEc, HCEs and HCEa algorithms are the best performers. This is one exception where the KM, SOM, and EM algorithms, and most notably the high performing FCM algorithm, do not do so well, especially in high classes or dimensions. As seen in the first column of Fig. (**6**), with any more than 4 classes these algorithms perform relatively poorly while the Euclidean distance based HC algorithms achieve a zero error rate regardless of the number of classes. This may be due to the difficulty in finding good initial seeds [12]. However, recall that the HCEs algorithm fails even at moderately low variances, and also the HCEa algorithm breaks down quickly as we increase the number of dimensions; see Fig. (**7**) where in almost all the plots the HCEs and HCEa algorithms approach an error rate worse than that of random labels.

Although the HCEc, HCEs, and HCEa algorithms perform well in low variance, the HCC algorithm should not be used in this case. In almost all of the graphs in Fig. (**5**), on the left edge of the plots where σ^2 ^= 0.1 this algorithm has a very high error rate while most of the other algorithms have nearly zero error. Also, HCC should be avoided when a high number of clusters is expected, as demonstrated in the third row of Fig. (**5**) where this algorithm is worse than random labels for all variances. However, given a small or moderate number of clusters (with high variance), the HCC algorithm can rival FCM, SOM, and KM; see the top row of Fig. (**5**).

For a fixed number samples, as we increase the number of clusters the EM and FCM algorithms tend to do very poorly. This is clear from the top row of Fig. (**6**). The situation seems to be a little better with more samples, as in the last row of Fig. (**6**). In the second and third columns of the same figure, note that in these moderate to high variances the KM algorithm does very well for any number of clusters. Meanwhile, FCM, EM, and SOM tend to do well only for a small number of clusters, and HCEc and HCEa do well when there are a large number of clusters.

In low dimensions, increasing the number of samples usually does not improve the error rate. See for example the top row of Fig. (**8**). However in higher dimensions, some clustering algorithms can use a large number of samples to overcome the difficulty of clustering with a large number of features. To see this effect, consider the middle column of Fig. (**8**). In the top plot we have *D* = 2 and here FCM has a nearly constant error rate for all *n*, at a little below 20%. In the next plot, we have *D* = 8 and we see that at least *n* = 200 samples are needed to achieve what appears to be an error floor a bit below 20%. Finally, in the third plot we have an extreme case with *D* = 128, where we need at least *n* = 2000 points, but with this many samples we can again achieve just below 20% error. In contrast, the HC and SOM algorithms seem to be poor at using additional samples to improve error rate. See the third row, middle column of Fig. (**8**), where all of the HC algorithms and the SOM algorithm actually have increasing error as the number of samples is increased, while the FCM and KM algorithms show significant improvement.

The EM algorithm improves with increasing sample size as well, in the safety of low variance or high dimensions. Though strangely, in higher variance situations a high number of samples appears to become a disadvantage. This phenomenon is illustrated in the upper right plot of Fig. (**8**) (*K* = 2, *D* = 2, σ^2 ^= 1), which reveals a weakness to large sample size, and in the third row, middle column of the same figure (*K* = 2, *D* = 128, σ^2 ^= 0.5), where it rivals the performance of FCM at the highest number of samples. Generally, the EM algorithm is excellent in high dimensions, especially in cases with low variance. This is seen clearly in the first and middle columns of Fig. (**7**).

## 6. CLUSTERING APPLIED TO GENOMICS

Data clustering has many applications, but in the last several years it has been applied increasingly to genomic studies and gene expression data [5, 6, 49, 15]. Each microarray slide can provide expression measurements for thousands of genes, and clustering is a useful exploratory technique to analyze this data. Through “guilt by association,” it can group similar genes together and aid biologists in identifying potentially meaningful relationships between them, while reducing the amount of information to analyze. Genes grouped together potentially have related functions or are co-regulated, as demonstrated by other evidence such as common promoter regulatory sequences and experimental verification. Often, there is the additional goal of identifying a small subset of genes that are most diagnostic of sample differences. Time-series clustering groups together genes whose expression levels exhibit similar behavior through time, with similarity considered suggestive of possible co-regulation.

Another common use of cluster analysis is the grouping of samples (arrays) by similarities in expression patterns. An expression pattern is effectively a complex phenotype, and clustering analysis is used to identify samples with similar and different phenotypes. In medical research, this approach allows the discrimination between pathologies based on differential patterns of gene expression, rather than relying on traditional histological methods. For instance, Eisen *et al*. [5] used cluster analysis to identify genes that show similar expression patterns over a wide range of experimental conditions in yeast.

The main assumption underlying unsupervised cluster analysis for gene expression data is that genes belonging to the same biological process or in the same pathway would have similar expression over a set of arrays (be it time-series or condition dependent). A large number of papers have been published describing algorithms for microarray data clustering [5, 8, 11, 50], but few analyze the relationship between the algorithms and the information that is supposed to be derived from the analysis [42]. In addition, these approaches are challenged by the large number of variables or genes to study, a limited understanding of the complete function of many genes, the small number of samples available, and a lack of knowledge in the underlying classes or subclasses.

Gene expression profiles refer to the expression values for a particular gene across various experimental conditions, or many genes under a single experimental condition. This distinction is a key point in the analysis to either reveal the responsiveness of genes (profiling), or discover new classes of genes for classification taxonomy. A great number of papers apply clustering algorithms to gene expression profiles, and in the following sections we provide examples of the two most common applications: the detection of co-expressed genes, and the discovery of sub-classes of diseases.

### 6.1. Detection of Co-Expressed Genes

Clustering genes is generally used to find groups of genes with similar expression, across either samples or time series, to generate hypotheses on the relationship between genes inside the same groups.

Johnson *et al*. [51] used a combination of statistical and clustering methodologies to define genomic profiles for early stages of the atherogenic response to benzo(a)pyrene, an environmental hydrocarbon that initiates oxidative stress in vascular smooth muscle cells. K-means, fuzzy cmeans, and hierarchical clustering were applied to genes found to be statistically significant (*via *ANOVA) to identify genes modulated by atherogenic insult in a redox-sensitive manner. These three non-supervised methods identified clones that were highly up-regulated by pro-oxidant alone, unaffected by antioxidant pretreatment, and neutralized by combined chemical treatments.

In [52], the authors proposed the use of Model Based Clustering [11] to group similar sequences in time series microarray data, with the goal of determining prototypes of expression showing patters, e.g., cyclic patterns. Using this technique, the authors were able to capture the qualitative behavior of time series data, grouping together genes with the same behavior.

### 6.2. Discovery of Sub-Classes of Diseases

Clustering techniques may be used to identify unrecognized tumor subtypes by, for example, applying a clustering algorithm to the samples in a set of data to group them based on similar gene expressions. If an initial partition agrees with prior biological understanding, further refining (sub-partitions) may reveal previously unknown sub-classes in cancer or other diseases. After a biological analysis of the validity of the newly discovered classes, they can then be used as input for the supervised training of a classifier used to derive improved prognoses based on molecular profiles. Some examples of this type of application are listed below.

In [53], the authors report the discovery of a subset of melanomas identified by clustering analysis of gene expression in a series of samples, and then identified genes that discriminate the groups.

The authors of [54] presented a study on the classification of human cancers for three adult cancer types: diffuse large B-cell lymphoma (DLBCL), follicular lymphoma (FL) and chronic lymphocyte leukemia (CLL). Their goal was to determine whether gene expression profiling could be used to find cancer sub-types as molecularly distinct diseases, with more homogeneous clinical behaviors. Hierarchical clustering and gene profiling facilitated the sub-classification of DLBCL into two groups, derived from different stages of B-cell differentiation and activation. In this case, the clustering process and visualization helped to create relevant hypotheses.

Two way hierarchical clustering was used in [55] to identify new molecular subtypes of acute myeloid leukemia (AML), including two prognostically relevant subgroups in AML with a normal karyotype. The unequal distribution of some mutations and morphologic subtypes between groups with different outcomes supported the concept that distinct biological changes may underlie the clinical phenotype. The authors also found that samples with two different mutations separate into different subgroups, which may lead to the identification of cooperating mutations and dysregulated pathways that eventuate in leukemogenesis.

In the same year, the authors of [56] used clustering on tumor samples to support the concept that parathyroid adenoma and hyperplasia are distinct entities with different molecular profiles.

Our final example is [57], where hierarchical clustering was applied to the microarray profile of 177 primary conventional renal cell carcinomas (cRCCs). The analysis segregated cRCC into five gene expression subgroups that correlated with survival in long-term follow up and was independent of grade, stage, and performance status. From the analysis, the authors identified a set of 259 genes that predict survival after surgery independently from clinical prognostic factors.

## 7. CONCLUSION

The choice of a clustering algorithm and a validation index is not a trivial one, more so when applying them to biological or medical high throughput data. Clustering algorithms should be chosen based on (a) the nature of the problem to solve (visualization, detection of sub-classes, etc.), (b) characteristics of the objects to be analyzed and the expected clusters, and (c) the size of the problem and computational power available. Some problems can be tackled by simple K-means clustering, while other situations may require more complex algorithms with larger memory or time requirements.

Regarding validation indices, in the absence of information to apply external validation, intuitively it might seem that relative indices should be more desirable than internal indices since they try to exploit data redundancy. However, most results have shown that even for simple models the relative indices do not give substantial improvement over the simpler internal indices, while at the same time potentially increasing computational costs beyond the limits of a desktop PC.

## Figures and Tables

**Fig. (1):**
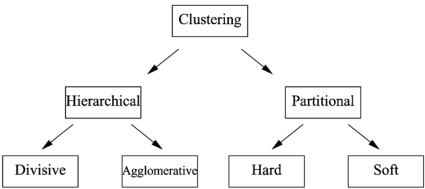
A basic taxonomy of clustering algorithms.

**Fig. (2):**
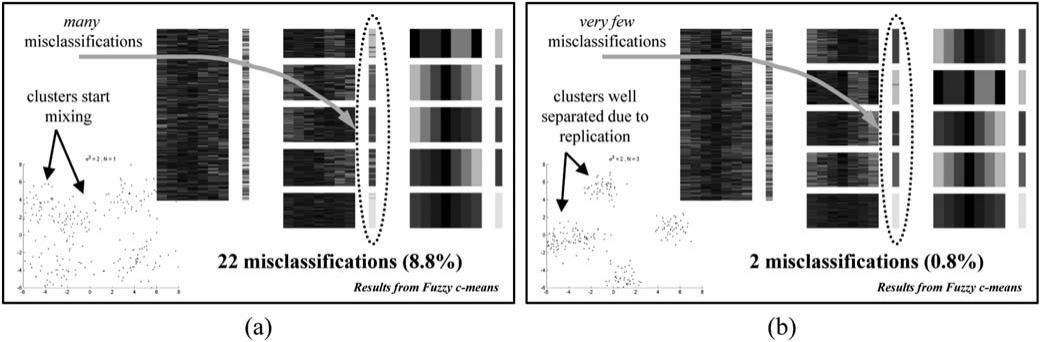
(**a**) Results from data simulated with high variability and no replicates. (**b**) Results from data simulated with high variability and three replicates.

**Fig. (3):**
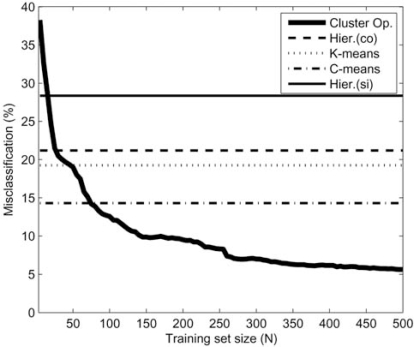
A comparison of the error of classical clustering algorithms *vs.* a trained cluster operator as a function of the number of training samples.

**Fig. (4):**
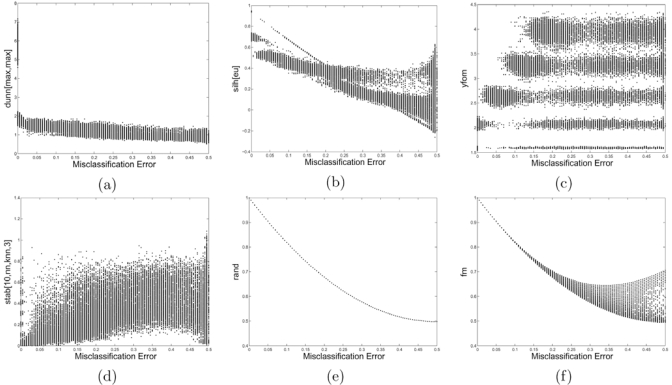
Scatter plots between some validation indices and the misclassification error. (**a**) Dunn's validity index, (**b**) Silhouette index with Euclidean distance, (**c**) Yeung's figure of merit, (**d**) Stability with 3NN, (**e**) Rand statistic, (**f**) Folkes and Mallows index.

**Fig. (5):**
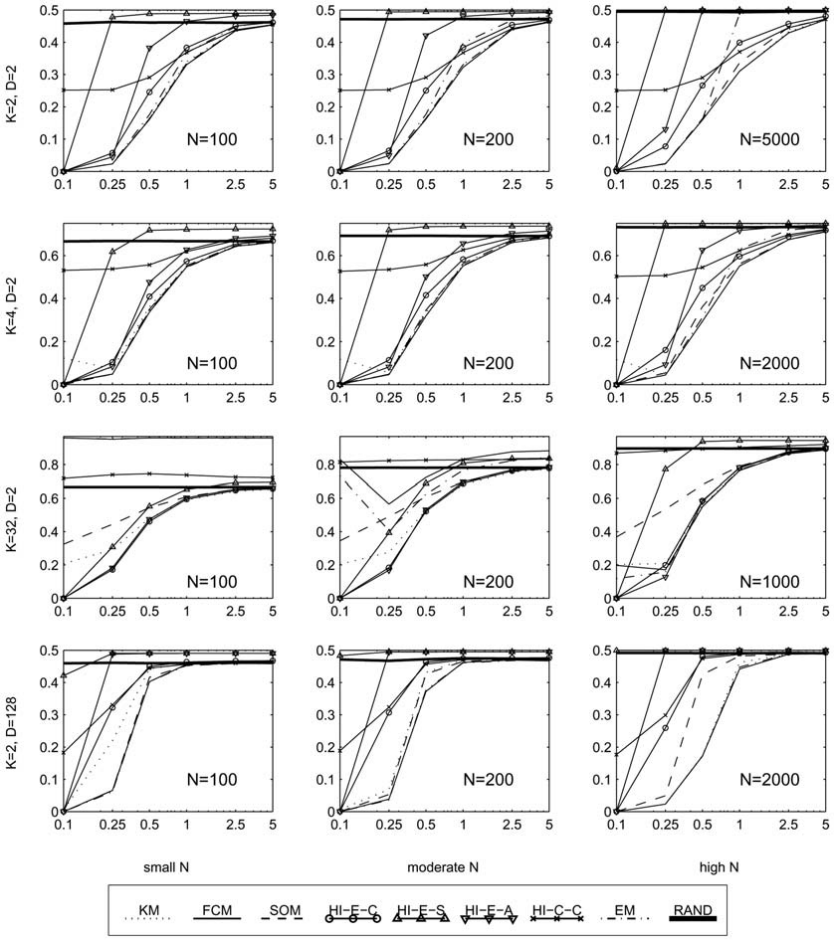
Performance with fixed templates and means in a planar grid, with respect to variance.

**Fig. (6):**
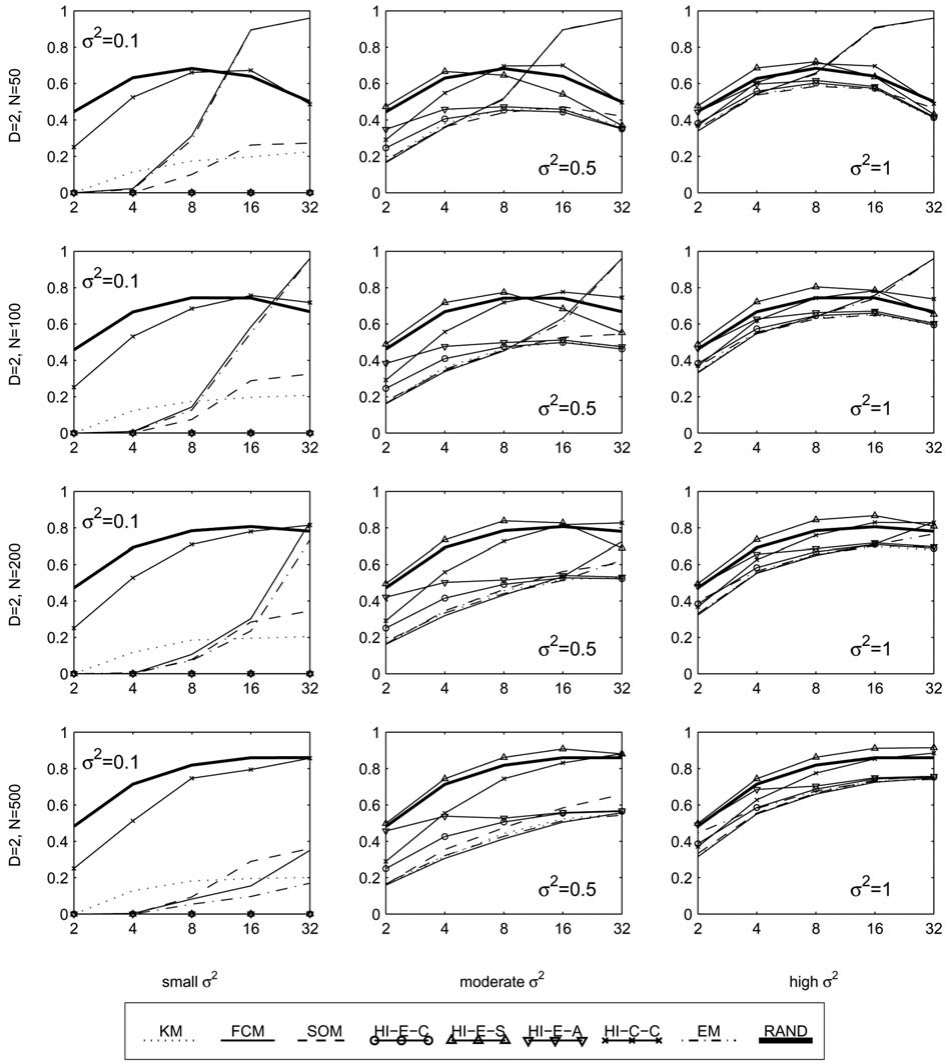
Performance with fixed templates and means in a planar grid, with respect to the number of clusters.

**Fig. (7):**
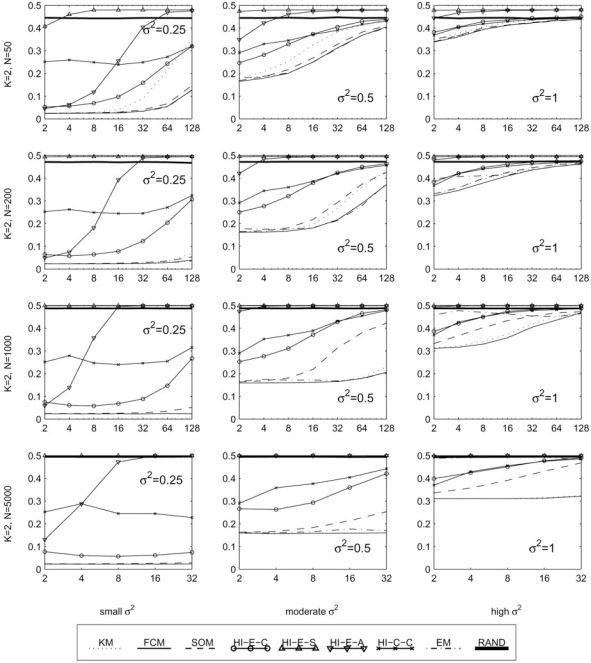
Performance with fixed templates and means in a planar grid, with respect to the number of dimensions.

**Fig. (8):**
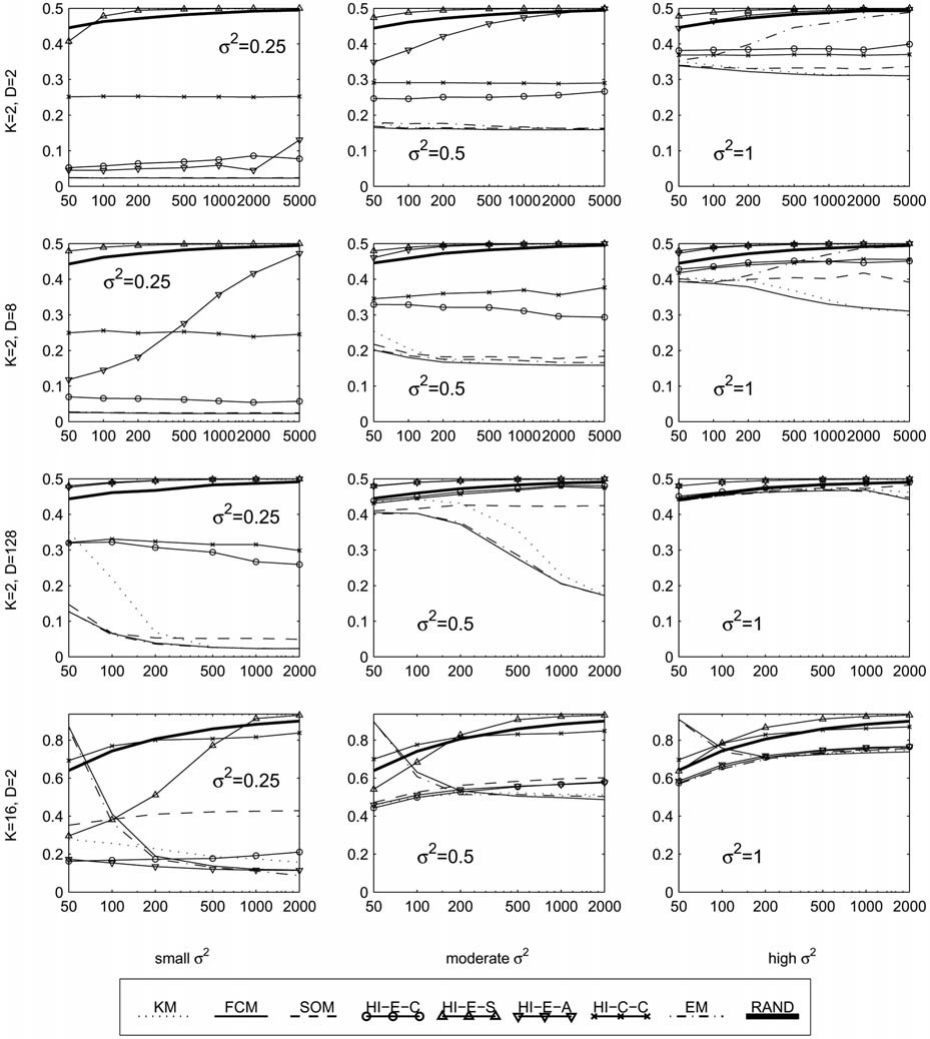
Performance with fixed templates and means in a planar grid, with respect to the total number of samples.

**Table 1:** Equations for Maximum Memory Usage in Each Clustering Algorithm and Three Examples

Algorithm	Memory Usage (in Bytes)	Exp. 1	Exp. 2	Exp. 3
KM	4*n* + 8*K*(2*D* + 1) + 72	8152	8712	12184
FCM	4*K*(6*n *+ 4*D *+ 3) + 114	96202	768818	100234
SOM	8*KD* + 132	164	388	2180
HC	16*n*^2^ + 40*n *+ 56	64080056	64080056	64080056
EM	4*K*(10*n *+ 12*D *+ 7) + 8*D*(*n* + 2*D *+ 2) + 214	192558	1314294	2484750

**Table 2:** Average Run Times for Three Representative Experiments, in Milliseconds

Variance	KM	FCM	SOM	HCEc	HCEs	HCEa	HCC	EM
**Experiment 1: *K *= 2, *D *= 2, *n *= 2000**								
0.25	0	0	20	4220	4280	4280	4360	0
1.0	0	20	0	4260	4180	4160	4260	140
5.0	0	80	0	4140	4140	4160		540
**Experiment 2: *K *= 16, *D *= 2, *n* = 2000**								
0.25	16	184	8	5072	5128	5496	5920	592
1.0	16	840	0	5816	5872	6216	6440	936
5.0	24	944	0	6512	7576	6936	6904	1216
**Experiment 3: *K *= 2, *D *= 128, *n* = 2000**								
0.25	142	114	114	5828	5800	5828	8057	742
1.0	142	171	114	5828	5828	5828	8057	5457
5.0	142	171	142	5828	5828	5828	8085	4828
